# Comparative analysis of PDE10A isoforms

**DOI:** 10.1186/1471-2210-11-S1-P56

**Published:** 2011-08-01

**Authors:** Corina Russwurm, Ronald Jäger, Doris Koesling, Michael Russwurm

**Affiliations:** 1 Institut für Pharmakologie und Toxikologie, Medizinische Fakultät, Ruhr-Universität Bochum, 44780 Bochum, Germany

## 

Phosphodiesterases (PDE) are considered to be key players in many signal transduction pathways. They degrade the second messengers cGMP and cAMP, thereby controlling their intracellular levels. So far, eleven PDE families, typically having several isoforms and splice variants are known. The PDE10 family is encoded by one gene that gives rise to at least two different isoforms in humans: the soluble PDE10A1 and the membrane associated PDE10A2. Distribution studies in human and mouse tissues showed highest expression in medium spiny neurons of the striatum and peripherally in testis. The localization of PDE10A in medium spiny neurons has led to much attention on PDE10 as a potential therapeutic target for novel antipsychotics. In neurons, membrane targeting of PDE10A2 is regulated at least in part by PKA-dependent phosphorylation of Thr16. On the other hand, little attention has been paid to PDE10A1 and the regulation of both isoforms.

Here, we compared PDE10A isoforms with regard to their tissue distribution, subcellular localization and biochemical properties.

